# AI-Based Characterization of Breast Cancer in Mammography and Tomosynthesis: A Review of Radiomics and Deep Learning for Subtyping, Staging, and Prognosis

**DOI:** 10.3390/cancers17203387

**Published:** 2025-10-21

**Authors:** Ana M. Mota

**Affiliations:** Instituto de Biofísica e Engenharia Biomédica, Faculdade de Ciências, Universidade de Lisboa, 1749-016 Lisboa, Portugal; ammota@fc.ul.pt

**Keywords:** breast cancer, artificial intelligence, mammography, breast tomosynthesis, radiomics, deep learning, tumor subtyping, tumor staging, prognosis

## Abstract

**Simple Summary:**

Breast cancer is the most common cancer in women. Today, treatment decisions still rely on biopsy, an invasive procedure that samples only a small part of the tumor and may not fully reflect its complexity. In recent years, artificial intelligence (AI) has shown promise in extracting biological and clinical information directly from standard breast images such as mammography and tomosynthesis. This review summarizes the latest research on how AI models based on radiomics and deep learning can predict tumor subtype, stage, and likely outcomes, without the need for tissue sampling. These tools could one day help personalize care, reduce invasive procedures, and make advanced diagnostics more accessible.

**Abstract:**

**Background**: Biopsy remains the gold standard for characterizing breast cancer, but it is invasive, costly, and may not fully capture tumor heterogeneity. Advances in artificial intelligence (AI) now allow for the extraction of biological and clinical information from medical images, raising the possibility of using imaging as a non-invasive alternative. **Methods**: A semi-systematic review was conducted to identify AI-based approaches applied to mammography (MM) and breast tomosynthesis (BT) for tumor subtyping, staging, and prognosis. A PubMed search retrieved 1091 articles, of which 81 studies met inclusion criteria (63 MM, 18 BT). Studies were analyzed by clinical target, modality, AI pipeline, number of cases, dataset type, and performance metrics (AUC, accuracy, or C-index). **Results**: Most studies focused on tumor subtyping, particularly receptor status and molecular classification. Contrast-enhanced spectral mammography (CESM) was frequently used in radiomics pipelines, while end-to-end deep learning (DL) approaches were increasingly applied to MM. Deep models achieved strong performance for ER/PR and HER2 status prediction, especially in large datasets. Fewer studies addressed staging or prognosis, but promising results were obtained for axillary lymph node (ALN) metastasis and pathological complete response (pCR). Multimodal and longitudinal approaches—especially those combining MM or BT with MRI or ultrasound—show improved accuracy but remain rare. Public datasets were used in only a minority of studies, limiting reproducibility. **Conclusions**: AI models can predict key tumor characteristics directly from MM and BT, showing promise as non-invasive tools to complement or even replace biopsy. However, challenges remain in terms of generalizability, external validation, and clinical integration. Future work should prioritize standardized annotations, larger multicentric datasets, and integration of histological or transcriptomic validation to ensure robustness and real-world applicability.

## 1. Introduction

Breast cancer remains the most commonly diagnosed cancer among women worldwide and is the leading cause of cancer-related mortality in this population [1]. The importance of early detection is well established, with multiple studies showing that cancers diagnosed at earlier stages are associated with significantly better outcomes [2].

When a suspicious lesion is detected on an imaging exam, typically mammography (MM), the patient is referred for additional tests, including further imaging and, imperatively, a biopsy to characterize the lesion at the tissue level. Core needle biopsy remains the gold standard for confirming malignancy, as it enables the extraction and histopathological analysis of a tissue sample from the lesion, often under image guidance.

While biopsy provides highly reliable diagnostic results, it presents several limitations. First, biopsies may provide an incomplete representation of the lesion, as the sampled tissue might not reflect the full heterogeneity of the tumor. Breast tumors often consist of multiple subclones and diverse cell populations, each potentially exhibiting different molecular or phenotypic profiles. Since the biopsy typically samples only a very small fraction of the lesion (sometimes as little as 0.0005% of the total tumor volume [3]), more aggressive or clinically relevant regions may be missed [4]. Second, biopsies are inherently invasive and frequently painful, often resulting in local trauma. Although these effects are typically temporary, some patients may experience longer-term complications or residual discomfort. Moreover, initial biopsy samples may produce inconclusive results, requiring additional procedures to ensure accurate pathological assessment. However, acquiring additional tissue may be challenging due to biopsy-induced alterations at the original site, potentially compromising subsequent histopathological evaluation [5]. Third, biopsies do not capture the surrounding tumor microenvironment, which plays a critical role in cancer progression and therapeutic response. This environment includes hypoxic regions, infiltrating immune cells and dynamic stromal interactions that may influence tumor aggressiveness, likelihood of invasion and resistance to treatment [6,7]. Lastly, biopsies are resource-intensive procedures as they require trained radiologists, specialized pathologists and advanced laboratory infrastructure. This makes them expensive and potentially less accessible in low-resource settings, where a higher threshold for biopsy referral may lead to delayed diagnosis and poorer clinical outcomes [8].

Medical imaging is the foundation of initial breast cancer detection. When considering breast imaging, there is a well-established set of modalities used in clinical practice: mammography (MM), breast tomosynthesis (BT), breast ultrasound (US) and magnetic resonance imaging (MRI). Among these, MM plays the central role, as it is the primary modality used in population-based screening programs. BT is increasingly used, particularly for women with dense breast tissue or for those at higher risk, and is progressively replacing MM in several screening settings [9,10]. In standard diagnostic workflows outside of organized screening, MM or BT is always the first imaging step, usually combined with US. MRI is generally used as a second-line imaging modality, depending on clinical findings, particularly in complex cases, for high-risk patients, or for pre-surgical planning. Therefore, imaging data is routinely available at the time of diagnosis.

In recent years, increasing attention has been directed toward utilizing these images not only for visual interpretation or basic quantitative assessment, but also as a rich source of complementary information about the lesion. This perspective has led to the emergence of advanced image analysis techniques, aiming to extract features that go beyond what is perceptible to the human eye. Among the most promising ideas is the hypothesis that certain genomic and proteomic patterns may be reflected in macroscopic imaging features, raising the possibility that imaging could serve as a non-invasive alternative or complement to tissue biopsy in the near future [11,12].

With this in mind, and driven by recent advances in computational power and the growing availability of data, interest in artificial intelligence (AI)-based methods has grown over the past few years. These approaches are based on the assumption that quantitative features extracted from imaging data may correlate with the molecular phenotype or genotype of a tumor. As a result, there has been a growing body of research exploring the potential of AI models to predict key aspects of tumor biology—such as tumor subtyping, tumor staging, and treatment response and prognosis—directly from imaging, potentially bypassing the need for invasive procedures.

Tumor subtyping and staging are currently determined through histopathological analysis of biopsy samples. In contrast, prediction of treatment response and prognosis is guided by predefined clinical protocols. Treatment response is typically assessed through longitudinal imaging studies, while prognosis is estimated based on a combination of clinical factors, including tumor subtype, size and stage. The prospect of inferring these critical parameters non-invasively from routine imaging data using AI-based approaches has opened new avenues for more accessible, patient-friendly workflows and earlier and more informative decision-making in breast cancer care.

Several existing reviews have explored the application of AI in breast cancer imaging, primarily focusing on lesion detection or segmentation and binary classification between benign and malignant findings [13,14]. However, despite the increasing volume of research in the last five years, reviews that specifically address the use of AI for biological and clinical characterization of breast tumors remain scarce, particularly those centered on MM and BT, which are the most widely used frontline imaging modalities in breast cancer detection.

The most relevant effort in this direction is the review by Elahi and Nazari [15], which provides a valuable overview of breast cancer characterization studies using various imaging modalities; however, it does not focus specifically on MM or BT. In contrast, Hussain et al. [16] present a comprehensive review on deep learning, radiomics and radiogenomics in breast image analysis, with a specific focus on BT and MM. Still, it does not include a dedicated analysis of studies addressing tumor biology prediction tasks.

The current review aims to fill that gap by providing a comprehensive overview of recent studies that employ AI to extract biological and prognostic information directly from MM and BT imaging data. It analyzes the clinical targets addressed, the datasets used, and the AI-based pipelines adopted (ranging from traditional radiomics to deep radiomics and end-to-end deep learning approaches). It includes a methodology section (Section 2) and an overview of AI-based pipelines for breast cancer characterization (Section 3). It then focuses on three core topics aligned with the clinical characterization pathway: tumor subtyping (Section 4.1), tumor staging (Section 4.2), and treatment response and prognosis (Section 4.3), followed by conclusions. Unless otherwise specified, the studies discussed refer to invasive breast carcinoma, which represents the most common histological subtype of breast cancer.

By focusing exclusively on biological and prognostic characterization tasks derived from standard mammography and tomosynthesis—the most widely available breast imaging modalities—this review offers a focused synthesis that bridges radiomics and deep learning approaches, providing a practical framework for clinical translation.

## 2. Methodology

**Figure 1:**
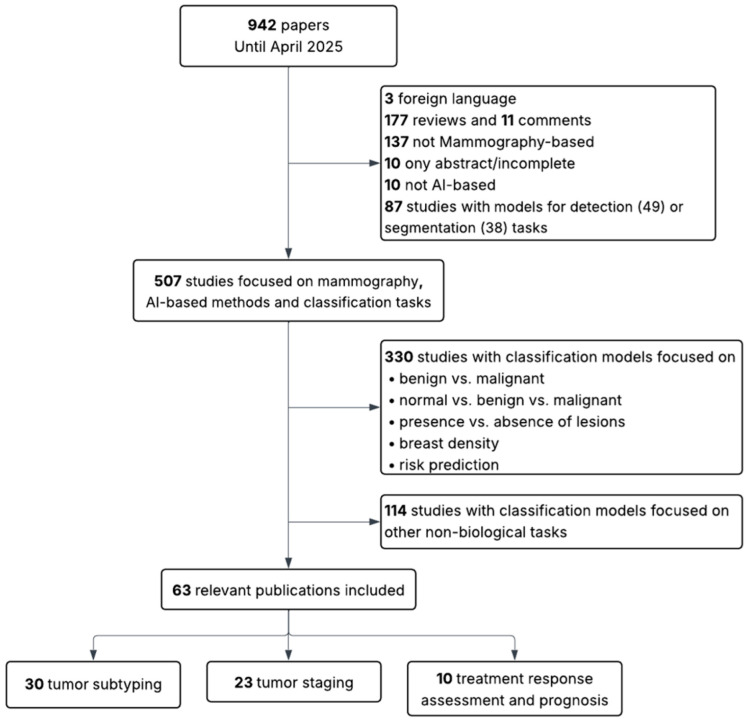
Flow diagram showing inclusion and exclusion criteria for MM-based studies.

**Figure 2:**
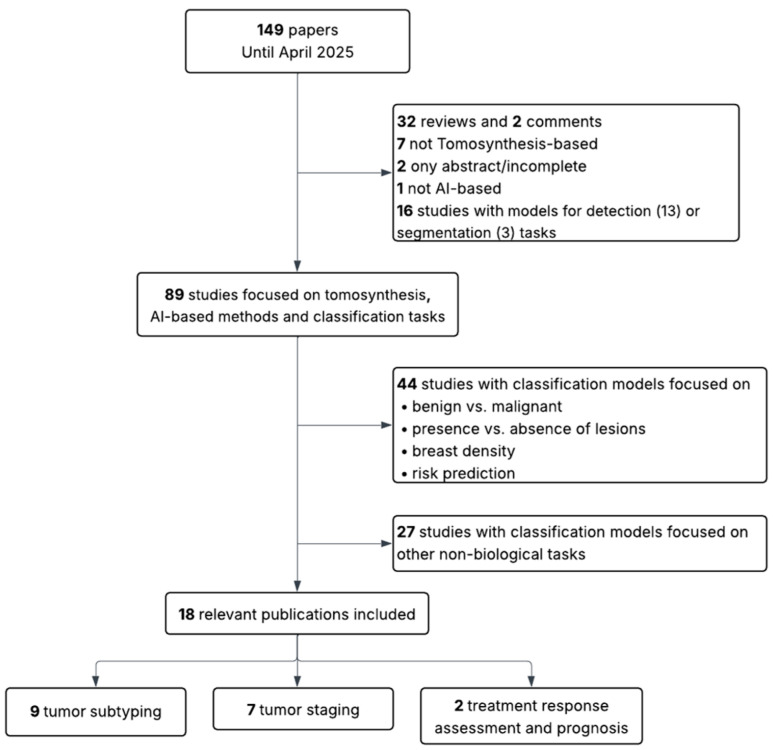
Flow diagram showing inclusion and exclusion criteria for BT-based studies.

This review followed a structured, semi-systematic literature search strategy to identify relevant studies addressing the use of AI for the biological and clinical characterization of breast tumors using MM or BT. A PubMed search was conducted using two separate queries: (“mammography” AND (“radiomics” OR “deep learning”)) and (“breast tomosynthesis” AND (“radiomics” OR “deep learning”)). Articles were screened based on title and abstract, and selection was conducted independently for each modality, as illustrated in Figure 1 and Figure 2.

The initial search yielded 942 articles for MM and 149 for BT. The same inclusion and exclusion criteria were applied to both sets. In the first stage, articles were excluded if they were not in English, if they were reviews, editorials, incomplete studies, or abstracts only. Studies that were not AI-based, not focused on MM or BT, or addressed detection or segmentation tasks rather than classification were also discarded. In the second stage, studies limited to binary classification tasks not directly related to the scope of this review (such as benign versus malignant lesion classification, presence or absence of lesions, breast density assessment, or general risk prediction) were excluded. Additionally, papers were excluded when their primary aim, despite involving classification, was the evaluation of other aspects such as radiomic stability, image quality, methodological validation, simulations, report generation, clinical performance assessment, or image reconstruction (in BT).

Following this process, 63 studies were retained for MM (30 addressing tumor subtyping, 23 staging, and 10 prognosis) and 18 studies for BT (9 addressing subtyping, 7 staging, and 2 prognosis). The MM group includes conventional mammography (MM), and contrast-enhanced spectral mammography (CESM).

A full list of included studies is presented in the following sections. They were systematically grouped into tables according to the following descriptors: clinical target, modality, AI approach, number of cases, type of dataset (public or private), and performance metric. Performance metrics considered were the area under the receiver operating characteristic curve (AUC, with 95% confidence intervals whenever available), accuracy, and in one case where both were absent, the concordance index (C-index). Several studies address more than one clinical target simultaneously.

## 3. AI-Based Pipelines Used in Breast Cancer Characterization

**Figure 3:**
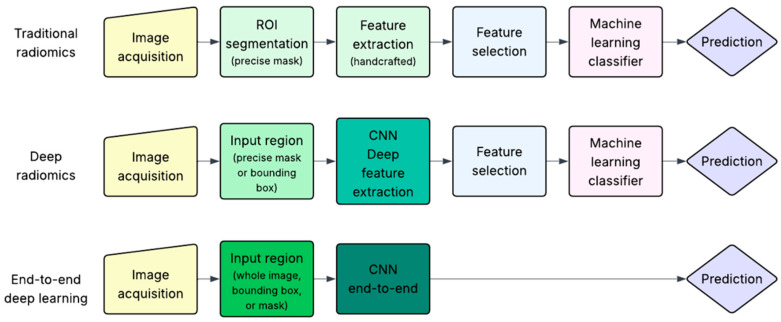
Overview of AI-based pipelines for breast cancer characterization. Traditional radiomics relies on handcrafted features from segmented ROIs. Deep radiomics used masks or bounding boxes and extracts deep features from Convolutional Neural Networks (CNNs). End-to-end DL predicts outcomes directly from the input region without explicit feature extraction.

As previously mentioned, this review focuses on studies that applied AI methods to extract biological characteristics of breast tumors from imaging data. These AI-based approaches can be broadly categorized into three groups: traditional radiomics, deep radiomics and end-to-end deep learning (DL), as illustrated in Figure 3.

All three approaches begin with image acquisition, an evident prerequisite for any image-based AI workflow. However, the subsequent steps differ substantially.

Traditional radiomics is based on the concept introduced in 2012 by Lambin et al. [11], in which a set of quantitative imaging features is extracted from medical images and analyzed to predict clinical targets. The key initial step is the precise segmentation of a region of interest (ROI), usually performed manually by radiologists, although semi-automatic and AI-assisted methods are increasingly available. This step is critical, as all subsequent analysis depends on the accuracy of the segmented area. Once the ROI is defined (typically as a binary mask), a large number of handcrafted features (e.g., intensity, texture, shape) are extracted. These are then passed through a feature selection step, which aims to retain only the most relevant and non-redundant features. Finally, a machine learning classifier is trained on the selected features to predict the target of interest (e.g., molecular subtype, stage, or prognosis).

Deep radiomics reduces some of the constraints of traditional radiomics, particularly regarding the segmentation step and the selection of features. Instead of a precise pixel-level mask, the information needed may be obtained through a broader region such as a bounding box surrounding the lesion. This region (either a precise mask or bounding box) is used as input to a convolutional neural network (CNN) to automatically extract deep features from specific intermediate layers of the network. These features are not predefined but rather learned directly from data. Because CNNs inherently learn hierarchical representations, explicit feature selection is often not necessary. These deep features are then passed to traditional machine learning classifier algorithms for prediction, similar to the traditional radiomics pipeline.

The end-to-end DL approach represents the most integrated but computationally intensive strategy. It can take as input the entire image, a bounding box, or a segmentation mask, thus offering greater flexibility and minimizing the need for precise lesion delineation. It uses CNNs which are trained to directly map input data to the desired prediction, learning both feature representations and classification boundaries simultaneously. Unlike deep radiomics, where features are extracted from intermediate layers, the end-to-end pipeline outputs the final classification or prediction directly, hence the term “end-to-end”. While powerful, this approach requires large, well-annotated datasets to avoid overfitting and achieve robust performance, a requirement that has only recently become more feasible with increased data availability and computational resources.

## 4. AI-Based Characterization of Breast Cancer from Mammography and Tomosynthesis

Following the description of the main AI-based pipelines, this section explores their application to clinically meaningful prediction tasks, grouped into three main categories: tumor subtyping, tumor staging and treatment response and prognosis.

**Figure 4:**
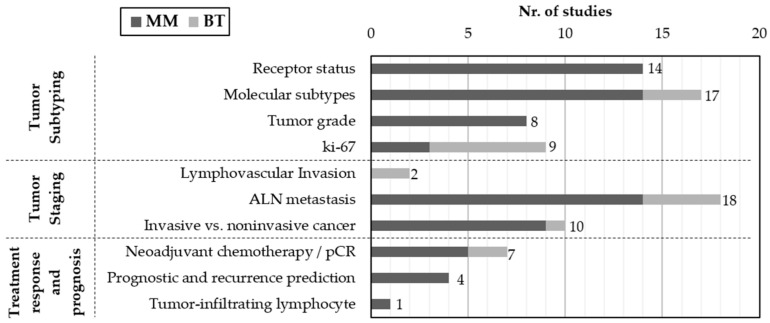
Distribution of studies by imaging modality (MM or BT) and clinical target. Studies were grouped into three main categories: tumor subtyping, tumor staging and treatment response/prognosis.

Figure 4 presents the distribution of studies according to the specific clinical targets addressed and imaging modalities (MM and BT). The majority of works are focused on tumor subtyping, particularly the prediction of molecular subtypes and receptor status, followed by ki-67expression and tumor grade. Staging-related tasks were also frequently reported, with emphasis on axillary lymph node (ALN) metastasis, followed by the distinction between invasive and in situ cancers and the identification of lymphovascular invasion. In contrast, fewer studies have addressed treatment response and prognosis, including the prediction of pathological complete response (pCR) to neoadjuvant chemotherapy and the estimation of recurrence risk. Only one study focused on the assessment of tumor-infiltrating lymphocytes.

In the following subsections, the studies are reviewed within three main categories—tumor subtyping, tumor staging, and treatment response and prognosis—reflecting key steps in clinical decision-making. Each category is accompanied by summary tables and a narrative discussion of methodological approaches, performance and clinical relevance.

### 4.1. Tumor Subtyping

Tumor subtyping refers to the stratification of breast cancer based on key biological markers that define the tumor’s molecular identity and clinical behavior. This includes the assessment of hormone receptor status (estrogen receptor (ER) and progesterone receptor (PR)), human epidermal growth factor receptor 2 (HER2) expression, ki-67proliferation index and tumor grade. With the exception of tumor grade, all other parameters determine the classification of tumors into molecular subtypes such as Luminal A (ER+, PR+, ki-67low, HER2−), Luminal B1 (ER+, PR− or ki-67high, HER2−), Luminal B2 (ER+, PR any, ki-67any, HER2+), HER2-enriched (ER−, PR−, ki-67any, HER2+) and Triple-Negative (ER−, PR−, ki-67any, HER2−), which are central to therapeutic decision-making and prognosis.

Subtyping is currently performed through histopathological analysis of biopsy samples, primarily using immunohistochemistry (IHC). However, recent studies suggest that imaging-based AI models may offer a non-invasive alternative. This section reviews studies that explore the use of MM and BT data to predict tumor subtype-related biomarkers, highlighting advances in receptor status classification, molecular subtype prediction, proliferation index estimation and tumor grading. Table 1 summarizes studies using MM- and BT-based imaging alone, while Table 2 lists those using multimodal approaches for breast cancer subtyping.

Among the reviewed works, radiomics-based pipelines remain dominant, particularly in CESM studies. La Forgia et al. (2020) [17] proposed a radiomics-based approach using CESM to predict multiple biomarkers, including ER, PR, HER2, Ki-67, tumor grade, and triple-negative (TN) status. Despite a small dataset (*n* = 52), the model yielded competitive AUCs of 0.8348 for HER2, 0.8213 for PR, and 0.9089 for TN. The pipeline employed manual lesion segmentation, 14 handcrafted features, and a leave one out cross-validation scheme. Although the risk of overfitting remains considerable given the sample size, the study illustrates the feasibility of multi-biomarker prediction from CESM.

DL-based approaches have also demonstrated strong performance, particularly for receptor status. Zhang et al. (2023) [18] developed a weakly supervised DL framework (BSNet) using a large MM dataset (*n* = 2321), achieving an AUC of 0.921 and accuracy of 0.802 for ER/PR classification. By training directly on full multi-view mammograms without manual ROIs and avoiding pixel-level annotations, BSNet supports scalability. It also provides visual feature maps that highlight relevant image regions, supporting interpretability. Duan et al. (2024) [19], using a smaller cohort (*n* = 358), implemented a ResNet-based architecture with attention mechanisms, achieving comparable performance. Similarly, it also generated attention maps highlighting suspicious tumor regions associated with ER status. These studies underscore that end-to-end DL pipelines can achieve robust results even without explicit lesion annotation, provided sufficient data is available.

For HER2 status prediction, Wang et al. (2025) [20] proposed a dual-modality framework combining MM and MRI data, combining handcrafted and deep features to differentiate HER2-zero, HER2-low, and HER2-positive tumor, aligning with the growing clinical importance of accurately identifying HER2-low patients for emerging targeted therapies. Their model achieved 0.8815 accuracy for HER2-positive detection and was validated across private and public datasets, including CMMD and Duke. The authors combined manual 3D tumor segmentation on MRI with automatic ResNet-50-based feature extraction on mammograms, followed by ComBat harmonization and synthetic minority oversampling technique (SMOTE) rebalancing. Though the exclusion of functional MRI sequences and semantic features is a limitation, the framework’s multimodal architecture and rigorous validation make it a benchmark in imaging-based HER2 classification.

In terms of molecular subtypes, Zhang et al. (2021) [21] used CESM-based radiomics to classify TN breast cancer. Following a univariate analysis and LASSO for feature selection, prediction models achieved a strong performance with an AUC of 0.90. On the other hand, Zhu et al. (2023) [22] explored a binary classification strategy to distinguish HER2-enriched vs. non-HER2-enriched, Luminal vs. non-Luminal and TN vs. non-TN subtypes using CESM. After feature selection with intraclass correlation coefficient (ICC) analysis, minimum Redundancy Maximum Relevance (mRMR) algorithm, and recursive feature elimination, support vector machine classifiers were trained for each task. The model performance was robust for HER2-enriched and Luminal tumors, but weaker for TN, reflecting a common challenge in capturing the heterogeneity of TN lesions. Importantly, their model incorporated clinical variables alongside radiomic features in a combined nomogram, providing a more clinically actionable framework. While their lower performance for TN may reflect class imbalance and subtle textural signs, the binary classification framework provides a pragmatic modelling strategy that mirrors how clinical decisions are often dichotomized, though its real-world applicability will require further validation in larger, balanced cohorts.

Other studies have approached the spatial heterogeneity of tumor signatures by analyzing not only the intratumoral region but also the surrounding peritumoral tissue for molecular subtype characterization. Niu et al. (2022) [23] analyzed intra- and peritumoral regions across MM, BT, and multiparametric MRI (T2WI, DCE, DWI). Despite a moderate sample (*n* = 241), the multimodal and multi-regional radiomic analysis led to strong predictive performance: AUCs of 0.938 for TN (DW + DCE) and 0.877 for HER2-enriched subtypes (DW + DCE). By constructing radiomics signatures separately for intra-, peri-, and combined regions in each modality, and comparing aggregated performances of DM + DBT versus DW + DCE, the study proposed a robust yet complex framework. While the methodology highlights the relevance of tumor microenvironment characterization, its reliance on advanced MRI sequences may limit immediate clinical translation, underscoring the need for simplified and effective pipelines.

Regarding Ki-67, Oba et al. (2024) [24] used DL on BT slices and achieved an AUC of 0.883 and accuracy of 0.912. Using Xception CNN as backbone, performance varied by lesion type, with better results in masses than in focal asymmetries or calcifications. The study also provided a nuanced analysis of preprocessing limitations, particularly how downsampling might obscure calcification detail. Jiang et al. (2022) [25] took a radiomics-based approach using MM, BT and MRI and analyzing intra- and peritumoral regions. The combined intra- and peritumoral radiomics signatures achieved a predictive performance with an AUC of 0.87, similar to Oba et al. Although their pipeline did not outperform the DL model from Oba et al., both studies suggest that ki-67 prediction is feasible using BT or multimodal imaging.

Tumor grade prediction has also been addressed. Mao et al. (2021) [26] used CESM-based radiomics to distinguish low- from high-grade tumors in a single-center study (*n* = 205), achieving an AUC of 0.80 and accuracy of 0.85. A two-step logistic regression model combined features from both low-energy and recombined CESM images, with the latter proving the most discriminative power, highlighting the role of contrast enhancement in grading. Petrillo et al. (2024) [27] extended the evidence provided by Mao et al.’s single-center CESM radiomics study to a multicenter setting, including four institutions. In this broader context, they reported an accuracy of 0.8205 for tumor grading using a decision tree classifier, thereby enhancing the generalizability. Marino et al. (2020) [28] compared CESM and MRI-based radiomics in 48 patients and showed that both modalities could effectively predict histological grade (accuracy > 0.77). While CESM outperformed MRI for hormone receptor status classification (95.6% vs. 82.6%), their performance in grading was comparable. This cross-modality comparison suggests that CESM could offer a cost-effective alternative to MRI for tumor grading, while also highlighting the value of combining imaging biomarkers from different modalities in future research.

In summary, tumor subtyping prediction has evolved from handcrafted radiomics applied to MM images toward increasingly sophisticated DL and multimodal pipelines. While radiomics remains dominant in MM and BT applications due to its interpretability and lower data demands, DL models, particularly weakly supervised or attention-based, have begun to demonstrate competitive performance with less reliance on manual annotation. Moreover, spatial and multi-region analyses are emerging as key strategies to capture tumor heterogeneity, especially for complex targets such as TN and Ki-67.

### 4.2. Tumor Staging

Tumor staging in breast cancer follows the TNM classification system, which integrates tumor size and extent of local invasion (T), presence of regional lymph node metastasis (N), and evidence of distant metastasis (M). Beyond size, a key histopathological concern is whether malignant cells have breached the basement membrane, invaded lymphatic or vascular structures, or disseminated to lymph nodes—factors that significantly impact prognosis and treatment decisions. Traditionally, these parameters are assessed through surgical excision, histological grading and sentinel lymph node biopsy. However, there is increasing interest in developing non-invasive alternatives based on imaging and artificial intelligence to infer these staging features preoperatively. Table 3 and Table 4 summarize the studies using MM- and BT-based AI models for tumor staging.

**Table 1:** Studies using mammography-based and tomosynthesis-based imaging for breast cancer subtyping.

Study	Clinical Target	Modality	AI Approach	No. of Cases	Dataset	Performance Metric
Zhou et al., 2019 [29]	Receptor status (HER2)	MM	TradRad	306 patients	private	AUC = 0.787 (95% CI, 0.673–0.885), Acc = 0.770
Ma et al., 2019 [30]	Molecular subtypes	MM	TradRad	182 patients	private	AUC = 0.865 (Acc = 0.796) TN vs. non-TN AUC = 0.784 (Acc = 0.748) HER2-enriched vs. non-HER2-enriched AUC = 0.752 (Acc = 0.788) luminal vs. non-luminal
Tagliafico et al., 2019 [31]	ki-67	BT (2D synthetic)	TradRad	70 patients	private	AUC = 0.698
La Forgia et al., 2020 [17]	Receptor status, tumor grade and ki-67	CESM	TradRad	52 patients	private	AUC = 0.8276 ER-positive vs. ER-negative AUC = 0.8213 PR-positive vs. PR-negative AUC = 0.8348 HER2-positive vs. HER2-negative AUC = 0.7985 ki-67+ vs. ki-67- AUC = 0.7680 High-grade vs. Low-grade AUC = 0.9089 TN vs. non-TN
Wang et al., 2020 [32]	Molecular subtype (TN vs. non-TN)	MM	TradRad	112 patients	private	AUC = 0.84 (95% CI, 0.73–0.96), Acc = 0.783
Marino et al., 2020 [33]	Receptor status and tumor grade	CESM	TradRad	100 patients	private	Acc = 0.784 HR-positive vs. HR-negative Acc = 0.972 HR-positive/HER2-negative vs. HR-negative/HER2-positive Acc = 1.00 HR-positive/HER2-positive (triple positive) vs. HR-negative/HER2-negative (TN) Acc = 0.821 TN vs. HR-positive Acc = 0.900 G1 vs. G2 + G3 for invasive cancers Acc = 1.00 G1 vs. G2 + G3 for non-invasive cancers
Son et al., 2020 [34]	Molecular subtypes	BT (2D synthetic)	TradRad	221 patients	private	AUC = 0.677 (95% CI, 0.552–0.802) Luminal vs. non-luminal AUC = 0.582 (95% CI, 0.361–0.804) HER2 vs. non-HER2 AUC = 0.868 (95% CI, 0.730–1.000) TN vs. non-TN
Kanbayti et al., 2021 [35]	Receptor status (PR)	MM	TradRad	184 patients	private	AUC = 0.652 (95% CI, 0.560–0.744) PR status
Ueda et al., 2021 [36]	Receptor status	MM	End-to-end DL	1373 images	private	AUC = 0.67 (95% CI, 0.58–0.76) ER-positive vs. ER-negative AUC = 0.61 (95% CI, 0.53–0.68) PR-positive vs. PR-negative AUC = 0.75 (95% CI, 0.68–0.82) HER2-positive vs. HER2-negative
Zhang et al., 2021 [21]	Molecular subtype (TN vs. non-TN)	CESM	TradRad	367 patients	private	AUC = 0.90 (95% CI, 0.85–0.96)
Mao et al., 2021 [26]	Tumor grade	CESM	TradRad	205 patients	private	AUC = 0.80 (95%CI, 0.67–0.92), Acc = 0.85
Ge et al., 2022 [37]	Molecular subtype (TN vs. non-TN)	MM	TradRad	319 patients	private	AUC = 0.809 (95% CI, 0.711–0.907), Acc = 0.806
Petrillo et al., 2022 [38]	Receptor status and tumor grade	CESM	TradRad	182 patients	private	AUC = 0.8237, Acc = 0.9167 G1 vs. G2 + G3 AUC = 0.7081, Acc = 0.8929 HER2-positive vs. HER2-negative AUC = 0.7500, Acc = 0.8500 HR-positive vs. HR-negative
Dominique et al., 2022 [39]	Tumor grade, ki-67, receptor status	CESM	End-to-end DL	389 patients	private	AUC = 0.621, Acc = 0.6028 G1 + G2 vs. G3 AUC = 0.858, Acc = 0.8125 ER-positive vs. ER-negative AUC = 0.630, Acc = 0.5958 PR-positive vs. PR-negative AUC = 0.644, Acc = 0.5544 HER2-positive vs. HER2-negative AUC = 0.610, Acc = 0.5645 Ki-67 (high or not) AUC = 0.908, Acc = 0.8468 TN
Jiang et al., 2022 [40]	ki-67	BT (slice-based)	TradRad and DeepRad	266 patients	private	AUC = 0.792 (95% CI, 0.647–0.897), Acc = 0.652 (95% CI, 0.404–0.913)
Zhang et al., 2023 [18]	Receptor status (ER and PR)	MM	End-to-end DL	2321 patients	private	AUC = 0.921, Acc = 0.802
Zhu et al., 2023 [22]	Molecular subtypes	CESM	TradRad	356 patients	private	AUC = 0.82 (95% CI, 0.69–0.92), Acc = 0.72 Luminal vs. Non-Luminal AUC = 0.83 (95% CI, 0.70–0.94), Acc = 0.83 HER2-enriched vs. Non-HER2-enriched AUC = 0.68 (95% CI, 0.47–0.86), Acc = 0.75 TN vs. Non-TN
Rong et al., 2023 [41]	Tumor grade	MM	TradRad	534 patients	private	AUC = 0.75 (95% CI, 0.66–0.83), Acc = 0.68 G3 vs. G1 + G2
Deng et al., 2024 [42]	Receptor status (HER2)	MM	TradRad	1512 patients	private	AUC = 0.724 (95% CI, 0.637–0.811), Acc = 0.722
Duan et al., 2024 [19]	Receptor status (ER)	MM	End-to-end DL	358 patients	private	AUC = 0.886 (95% CI, 0.809–0.934), Acc = 0.831
Petrillo et al., 2024 [27]	Receptor status, molecular subtype (luminal vs. non-luminal) and tumor grade	CESM	TradRad	169 patients	private	Acc = 0.6071 HER2-positive Acc = 0.6786 HR-positive Acc = 0.8205 grade Acc = 0.9375 luminal vs. non-luminal
Mota et al., 2024 [43]	Molecular subtypes	MM	End-to-end DL	660 patients	semiPublic—OPTIMAM	AUC = 0.6599, Acc = 0.7372 Luminal A vs. non-Luminal A AUC = 0.6545, Acc = 0.7491 Luminal B1 vs. non-Luminal B1 AUC = 0.6530, Acc = 0.6284 HER2 vs. non-HER2, AUC = 0.6445, Acc = 0.7706 TN vs. non-TN Multiclassification: AUC = 0.5992 Luminal A AUC = 0.5981 Luminal B1 AUC = 0.5920 Luminal B2 AUC = 0.6440 HER2 AUC = 0.6089 TN
Bakker et al., 2024 [44]	Molecular subtypes	MM	TradRad	186 patients	semipublic-OPTIMAM	AUC = 0.855 (95% CI, 0.779–0.930), Acc = 0.815 luminal A AUC = 0.812 (95% CI, 0.736–0.889), Acc = 0.734 luminal B AUC = 0.755 (95% CI, 0.644–0.867), Acc = 0.637 HER2 AUC = 0.789 (95% CI, 0.701–0.878), Acc = 0.718 TN
Nissar et al., 2024 [45]	Molecular subtypes	MM	End-to-end DL	749 patients	public—CMMD	Subtype classification (4-class: Luminal A, Luminal B, HER2-enriched, TN): Acc = 0.90 (mass-based) Acc = 0.90 (calcification-based) Acc = 0.92 (combined mass + calcification)
Oba et al., 2024 [24]	ki-67	BT (slice-based)	End-to-end DL	126 patients	private	AUC = 0.883, Acc = 0.912 AUC = 0.890 mass AUC = 0.750 calcification AUC = 0.870 distortion AUC = 0.660 focal asymmetric density
Zeng et al., 2025 [46]	Receptor status	MM	End-to-end DL	352 patients	private	AUC = 0.708 (95% CI, 0.609–0.808), Acc = 0.651 HER2 status AUC = 0.785 (95% CI, 0.673–0.897), Acc = 0.845 ER status AUC = 0.706 (95% CI, 0.603–0.809), Acc = 0.678 PR status
Rabah et al., 2025 [47]	Molecular subtypes	MM	End-to-end DL	1775 patients (public)	public—CMMD	AUC = 0.8887, Acc = 0.6379 (multiclass classification) AUC = 0.67 Luminal A vs. Others AUC = 0.74 Luminal B vs. Others AUC = 0.78 HER2 vs. Others AUC = 0.78 TN vs. Others
Hu et al., 2025 [48]	ki-67	BT (slice-based)	TradRad	289 patients	private	AUC = 0.755 (95% CI, 0.629–0.880), Acc = 0.782
Mota et al., 2025 [49]	Molecular subtypes	BT (slice-based)	End-to-end DL	453 images	semiPublic—OPTIMAM	AUC = 0.5928 Luminal B2 vs. non-Luminal B2 AUC = 0.7317 HER2+ vs. non-HER2+ AUC = 0.6522 TN vs. non-TN

**Table 2:** Studies using multimodal imaging approaches (including mammography and tomosynthesis) for breast cancer subtyping.

Study	Clinical Target	Modality	AI Approach	No. of Cases	Dataset	Performance Metric
Marino et al., 2020 [28]	Receptor status and tumor grade	CESM and MRI	TradRad	48 patients	private	CESM: Acc = 0.956 HR positive vs. HR negative Acc = 0.778 G1 + G2 vs. G3 invasive cancers MRI: Acc = 0.826 HR positive vs. HR negative Acc = 0.778 G1 + G2 vs. G3 cancers invasive cancers
Jiang et al., 2022 [25]	ki-67	MM and MRI and BT (slice-based)	TradRad	209 patients	private	AUC = 0.866
Niu et al., 2022 [23]	Molecular subtypes	MM and MRI and BT (slice-based)	TradRad	241 patients	private	AUC = 0.773 (95% CI, 0.650–0.895) Luminal A (MM + BT) AUC = 0.747 (0.578–0.917) Luminal A (DW + DCE) AUC = 0.807 (0.706–0.908) Luminal B (MM + BT) AUC = 0.784 (0.675–0.894) Luminal B (DW + DCE) AUC = 0.802 (0.658–0.946) HER2 (MM + BT) AUC = 0.877 (0.795–0.959) HER2 (DW + DCE) AUC = 0.874 (0.759–0.990) TN (MM + BT) AUC = 0.938 (0.883–0.992) TN (DW + DCE)
Liu et al., 2023 [50]	Molecular subtype (Luminal A vs. non-Luminal A)	MM and MRI	End-to-end DL	158 patients	private	AUC = 0.802 (95% CI, 0.657–0.906), Acc = 0.711 (MM + MRI) AUC = 0.593 (95%CI, 0.436–0.737), Acc = 0.533 (only MM)
Zhang et al., 2023 [51]	Molecular subtypes	MM and US	End-to-end DL	4162 images	private	AUC = 0.929 (95% CI, 0.903, 0.951), Acc = 88.5 (86.0–90.9) Luminal (Luminal A and Luminal B) vs. Non-Luminal (HER2-enriched and TN)
Liu et al., 2024 [52]	ki-67	BT (slice-based) and US	TradRad	149 patients	private	AUC = 0.818 (95% CI, 0.685–0.950)
Wang et al., 2025 [20]	Receptor status (HER2)	MM and MRI	TradRad and DeepRad	550 patients (private)	private and public (4validation)—Duke, CMMD	AUC = 0.824 (95% CI, 0.749–0.884), Acc = 0.8074, HER2-Positive vs. HER2 Zero/Low AUC = 0.811 (95% CI, 0.735–0.874), Acc = 0. 7926, HER2-Zero vs. HER2-Low/Positive Acc = 0.8444 (HER2-zero) Acc = 0.8000 (HER2-low) Acc = 0.8815 (HER2-positive)
Yang et al., 2025 [53]	Molecular subtypes	MM and MRI	TradRad	243 patients	private	AUC = 0.648, Acc = 0.627 Luminal A vs. Luminal B AUC = 0.819, Acc = 0.793 luminal A vs. HER2-enriched AUC = 0.725, Acc = 0.696 luminal A vs. TN AUC = 0.644, Acc = 0.560 luminal B vs. HER2-enriched AUC = 0.625, Acc = 0.636 luminal B vs. TN AUC = 0.598, Acc = 0.500 TN vs. HER2-enriched

Ductal carcinoma in situ (DCIS) progression to invasive carcinoma has been a major focus. Alaeikhanehshir et al. (2024) [54] investigated whether DL applied to MM could distinguish low- from high-risk DCIS, aiming to guide eligibility for active surveillance. Using a fully convolutional network based on U-Net trained on mammograms from 464 patients with preoperative DCIS, the study achieved an AUC of 0.72 for distinguishing low- from high-grade DCIS, which increased to 0.76 when cases upstaged to invasive cancer were also considered high-risk. Although the performance was modest, the study addresses a clinically pressing challenge—avoiding overtreatment of indolent DCIS—and reports a high negative predictive value (0.91), supporting the model’s utility in safely identifying low-risk cases. As one of the few AI studies focused on the transition from in situ to invasive disease, it contributes meaningfully to efforts toward personalized, less invasive management of DCIS, even if further validation is needed before clinical translation.

Wu et al. (2024) [55] extended the investigation of DCIS staging using a larger cohort (*n* = 733) and a multimodal input strategy combining US with MM, clinical information, and core needle biopsy pathology. Their end-to-end DL framework achieved strong performance across multiple classification tasks, with AUCs ranging from 0.859 to 0.907 for the three-class grading task (low-, intermediate/high-grade, upstaged DCIS), from 0.829 to 0.861 for DCIS versus upstaged DCIS, and up to 0.987 for distinguishing low-grade from upstaged low-grade DCIS, with accuracies reaching 0.939 Notably, the model was evaluated across different nuclear grades and histological presentations, including the challenging task of distinguishing low-grade DCIS from upstaged low-grade DCIS. This stratified analysis enhances clinical relevance by addressing uncertainty in DCIS progression risk. The model’s high performance may be partly attributable to the added diagnostic value of US, which improves visualization of soft tissue. The absence of external validation and cohort-specific performance analysis, however, leaves questions about generalizability.

In a related study, Wu et al. (2025) [56] focused on the preoperative prediction of low nuclear grade DCIS using traditional radiomics in a smaller cohort (*n* = 237). Their ensemble model—built using Elastic Net, Generalized Linear Models with Boosting, and Ranger—achieved an AUC of 0.92 and accuracy of 0.88 in the validation set, outperforming a baseline model based solely on clinical and conventional imaging variables. Importantly, the authors applied Shapley Additive Explanations (SHAP) analysis to interpret the contribution of individual radiomic features, emphasizing that transparency and interpretability can be preserved while maintaining high performance. Although limited by its modest sample size and lack of external validation, the integration of radiomic features with clinical information supports the role of interpretable radiomics in preoperative decision-making for DCIS.

Invasiveness assessment has also been addressed using CESM and MRI. Marino et al. (2020) [28] evaluated the ability of radiomics applied to both CESM and MRI to differentiate invasive from non-invasive breast cancers in a small cohort (*n* = 48), reporting an accuracy of 0.92 for CESM and 0.90 for MRI. In an earlier study with 100 patients [33], the same group had already demonstrated solid performance using CESM alone (accuracy = 0.874). The consistency across studies supports the hypothesis that vascular enhancement patterns captured by CESM are highly informative for assessing invasive potential within preoperative DCIS cases. These findings reinforce the role of CESM not only in lesion detection but also as a functional imaging tool for early biological characterization.

Axillary lymph node (ALN) metastasis has emerged as the most studied clinical target in recent AI-based breast cancer staging research, reflecting its central role in treatment decisions such as axillary dissection or systemic therapy. Several studies have explored this using both DL and traditional radiomics. Abel et al. (2022) [57] developed an end-to-end DL model based on MM alone and achieved a remarkable accuracy of 0.9596 for suspicious lymph nodes in a small sample size (*n* = 74). This work stands out by showing that even conventional MM alone can carry predictive signals for ALN status. However, the absence of external validation and small, homogeneous cohorts limit clinical applicability. Xu et al. (2023) [58] adopted a slice-based radiomics strategy on BT and achieved an AUC of 0.92 and accuracy of 0.833 in 120 patients. While radiomics may offer greater interpretability and model transparency, its sensitivity to segmentation protocols and image preprocessing requires caution when comparing across datasets.

Larger studies by Wang et al. (2024) [59] and Hua et al. (2024) [60] combined MM and MRI for ALN prediction, achieving consistent results (AUC ~0.90, accuracy ~0.82–0.85) in cohorts of nearly 500 patients. These multimodal pipelines emphasize the value of integrating structural and functional data when targeting complex endpoints such as lymphatic spread. However, both studies lack stratification by tumor subtype or size, which may influence nodal involvement patterns. They also underscore a practical limitation of MRI availability, which may restrict model implementation in standard clinical settings.

As for lymphovascular invasion, Wang et al. (2022) [61] and Xu et al. (2024) [62] proposed two different BT-based radiomics strategies. Wang et al. used a volumetric approach with 3D features extracted from the entire tumor volume, achieving an AUC of 0.835 in a cohort of 135 patients. Xu et al., using slice-based 2D feature extraction from intra- and peritumoral regions, reported superior performance (AUC = 0.905, Acc = 0.815) in a slightly larger cohort (*n* = 178). The higher performance of the latter may stem from differences in ROI selection, feature robustness, or even cohort composition. Both studies underscore the value of addressing lymphovascular invasion non-invasively, a target rarely explored in AI-based breast cancer imaging research, despite its clinical relevance. Moreover, they demonstrate how BT can provide meaningful 3D tissue characterization, particularly in detecting subtle signs of microinvasion.

Overall, AI has shown to be able of assisting in complex staging tasks, from detecting occult invasion to predicting nodal or vascular involvement. While CESM shows potential for evaluating invasiveness, and BT demonstrates value for lymphovascular invasion detection, the most consistent gains are observed when multimodal data or sophisticated DL architectures are used.

**Table 3:** Studies using mammography-based and tomosynthesis-based imaging for breast cancer staging.

Study	Clinical Target	Modality	AI Approach	No. of Cases	Dataset	Performance Metric
Shi et al., 2018 [63]	Pure DCIS vs. DCIS with invasion	MM	TradRad and DeepRad	99 patients	private	TradRad: AUC = 0.68 (95% CI, 0.66–0.71) DeepRad: AUC = 0.70 (95% CI, 0.68–0.73)
Yang et al., 2019 [64]	ALN metastasis	MM	TradRad	147 patients	private	AUC = 0.875 (95% CI, 0.698–0.891), Acc = 0.800 (95% CI, 66.4–83.2%)
Li et al., 2019 [65]	Pure DCIS vs. DCIS with invasion	MM	TradRad	362 patients	private	AUC = 0.72 (95% CI, 0.63–0.81)
Tan et al., 2020 [66]	ALN metastasis	MM	TradRad	216 patients	private	AUC = 0.863 (95% CI, 0.821–0.897), Acc = 0.7917
Mao et al., 2020 [67]	ALN metastasis	CESM	TradRad	394 patients	private	AUC = 0.767 (95% CI, 0.583–0.857) internal validation AUC = 0.790 (95% CI, 0.63–0.94) external validation
Marino et al., 2020 [33]	Pure DCIS vs. DCIS with invasion	CESM	TradRad	100 patients	private	Acc = 0.874 invasive vs. noninvasive cancer
Kanbayti et al., 2021 [35]	ALN metastasis	MM	TradRad	184 patients	private	AUC = 0.681 (95% CI, 0.559–0.804)
Wu et al., 2022 [68]	ALN metastasis	CESM	DeepRad	182 patients	private	for non-sentinel lymph node metastasis status: AUC = 0.85 (95% CI, 0.71–0.99), Acc = 0.81 (95% CI, 0.63–0.93) testing AUC = 0.82 (95% CI, 0.67–0.97), Acc = 0.74 (95% CI, 0.55–0.88) temporal validation axillary tumor burden: AUC = 0.82 (95% CI, 0.66–0.97), Acc = 0.75 (95% CI, 0.55–0.88) testing AUC = 0.77 (95% CI, 0.62–0.93), Acc = 0.74 (95% CI, 0.55–0.88) temporal validation
Abel et al., 2022 [57]	ALN metastasis	MM	End-to-end DL	74 patients	private	Acc = 0.9596
Hou et al., 2022 [69]	Pure DCIS vs. DCIS with invasion	MM	TradRad	700 patients	private	AUC = 0.71 (95% CI, 0.62–0.79)
Wang et al., 2022 [61]	Lymphovascular Invasion	BT (volume-based)	TradRad	135 patients	private	AUC = 0.835 (95% CI, 0.712–0.958)
Lin et al., 2023 [70]	ALN metastasis	CESM	TradRad	365 patients	private	AUC = 0.7567 (95% CI, 0.6717–0.8678), Acc = 0.7551 (0.6113–0.8666)
Wang et al., 2023 [71]	ALN metastasis	CESM	TradRad	809 patients	private	AUC = 0.732, Acc = 0.681
Xu et al., 2023 [58]	ALN metastasis	BT (slice-based)	TradRad	120 patients	private	AUC = 0.920 (95% CI, 0.806–1.000), Acc = 0.833
Shimokawa et al., 2023 [72]	Stromal invasion/pure DCIS vs. DCIS with invasion	BT (slice-based)	End-to-end DL	140 patients	private	AUC = 0.75 (95% CI, 0.69–0.81)
Tsai et al., 2024 [73]	Pure DCIS vs. DCIS with invasion	MM	End-to-end DL	1436 patients	private	AUC = 0.747 (95% CI, 0.677–0.813)
Alaeikhanehshir et al., 2024 [54]	Pure DCIS vs. DCIS with invasion	MM	End-to-end DL	464 patients	private	AUC = 0.72 low-risk DCIS vs. high risk AUC = 0.76 low-risk DCIS vs. high-risk and/or upstaged DCIS (occult IBC)
Xu et al., 2024 [62]	Lymphovascular Invasion	BT (slice-based)	TradRad	178 patients	private	AUC = 0.905 (95% CI, 0.823–0.986), Acc = 0.815
He et al., 2025 [74]	ALN metastasis	BT (volume-based)	TradRad	536 patients	private	AUC = 0.792, Acc = 0.726

**Table 4:** Studies using multimodal imaging approaches (including mammography and tomosynthesis) for breast cancer staging.

Study	Clinical Target	Modality	AI Approach	No. of Cases	Dataset	Performance Metric
Marino et al., 2020 [28]	Pure DCIS vs. DCIS with invasion	CESM + MRI	TradRad	48 patients	private	CESM Acc = 0.92 MRI Acc = 0.90
Cheng et al., 2022 [75]	ALN metastasis	MM + MRI + BT (slice-based)	TradRad	208 patients	private	AUC = 0.786, Acc = 0.771 (MM + BT) AUC = 0.826, Acc = 0.829 (DCE-MRI + DWI)
Haraguchi et al., 2023 [76]	ALN metastasis	MM + BT (2D synthetic)	TradRad	77 patients	private	AUC = 0.738 (95% CI, 0.608–0.867), MM AUC = 0.742 (95% CI, 0.613–0.871), BT (2D synthetic)
Wang et al., 2024 [59]	ALN metastasis	MM + MRI	TradRad	485 patients	private	AUC = 0.892 (95% CI, 0.826–0.939), Acc = 0.8182
Hua et al., 2024 [60]	ALN metastasis	MM + MRI	TradRad	492 patients	private	AUC = 0.902 (95% CI, 0.833–0.972), Acc = 0.847
Guo et al., 2024 [77]	ALN metastasis	MM + MRI	TradRad and DeepRad	270 patients	private	AUC = 0.846, Acc = 0.765
Wu et al., 2024 [55]	Pure DCIS vs. DCIS with invasion	MM + US	End-to-end DL	733 patients	private	AUC = 0.859–0.907, Acc = 0.752–0.766 in low-grade DCIS vs. intermediate-to-high-grade DCIS vs. upstaged DCIS AUC = 0.829–0.861, Acc = 0.751–0.780 in DCIS vs. upstaged DCIS AUC = 0.769–0.987, Acc = 0.818–0.939 in low-grade DCIS vs. upstaged low-grade DCIS
Cheng et al., 2025 [78]	ALN metastasis	MM + MRI	TradRad and End-to-end DL	326 patients	private	AUC = 0.877, Acc = 0.727
Wu et al., 2025 [56]	Pure DCIS vs. DCIS with invasion	MM + US	TradRad	237 patients	private	AUC = 0.92 (95% CI, 0.84–0.96), Acc = 0.88 low nuclear grade DCIS vs. Intermediate to high nuclear grade

### 4.3. Treatment Response and Prognosis

Breast cancer management is increasingly shaped by the ability to anticipate treatment efficacy and long-term outcomes. In this context, AI offers a non-invasive path to predict clinically meaningful endpoints such as pathological complete response (pCR) to neoadjuvant chemotherapy, recurrence risk, and immunological markers like tumor-infiltrating lymphocytes. Predicting pCR preoperatively can help tailor therapy intensity and avoid overtreatment, while recurrence risk models may guide follow-up strategies and adjuvant therapy decisions. The studies summarized in Table 5 and Table 6 reflect a growing interest in integrating AI into therapeutic planning and outcome prediction, though their clinical translation remains limited by methodological challenges and dataset constraints.

Mao et al. (2022) [79] employed CESM-based radiomics to predict pCR in 118 breast cancer patients receiving neoadjuvant chemotherapy. Their pipeline integrated intratumoral and peritumoral features, achieving an AUC of 0.85. This inclusion of peritumoral regions reflects an emerging recognition that tumor-stroma interactions influence therapeutic outcomes. The model, based on multivariate logistic regression, offers interpretability while maintaining competitive performance. A notable strength lies in the detailed radiomic feature selection and the clarity of the biological rationale, particularly the correlation of peritumoral enhancement with response. However, the study would benefit from a broader validation across imaging centers to assess robustness.

Xing et al. (2024) [80] adopted a different approach to predict pCR in ER+/HER2− patients undergoing neoadjuvant chemotherapy, integrating CESM with an end-to-end DL model. In a cohort of 265 patients, the model achieved excellent results (AUC = 0.95; Acc = 0.94), by integrating deep features from low-energy and recombined CESM images with selected clinical and pathological variables. The study included a nomogram incorporating these fused inputs, offering a more interpretable and potentially deployable tool for clinical use. The inclusion of an independent test set lends credibility to the performance metrics, which are often overestimated in small internal validations. However, the restriction to a single molecular subtype (ER+/HER2−) raises questions about generalizability, especially since neoadjuvant chemotherapy response is known to vary substantially across subtypes. While the study presents a strong case for CESM-based DL in the pCR setting, broader validation across subtypes and imaging centers remains a necessary next step.

Förnvik et al. (2024) [81] explored the potential of temporal modeling using volume-based BT images to predict pCR in a prospective NAC setting. The study stands out methodologically by integrating volumetric changes across different timepoints of chemotherapy, simulating a longitudinal assessment of treatment response. A 3D ResNet with an attention module was trained on volumetric inputs from three timepoints of chemotherapy, simulating a longitudinal assessment of treatment response, rather than relying on static pre-treatment imaging. Despite a relatively modest sample size (*n* = 149), the model achieved an AUC of 0.83, highlighting the added value of temporal information. GradCAM analyses showed that the network consistently focused on similar tumor regions across timepoints, suggesting that it captured relevant treatment dynamics. However, BT’s limited soft tissue contrast compared to CESM or MRI may reduce its ability to detect more subtle changes in tumor microenvironment, possibly constraining its utility in borderline or heterogeneous responses.

Beyond response prediction, several studies addressed recurrence and long-term prognosis. Jiang et al. (2020) [82] developed a MM-based radiomics model to construct a nomogram for predicting invasive disease-free survival in patients with triple-negative breast cancer. From 136 extracted features, a 14-feature Rad-score was derived using LASSO and incorporated with pathological nodal stage into a multivariate regression nomogram. This combined model achieved a C-index of 0.944 in the validation cohort, indicating strong prognostic discrimination and outperforming a clinical model based solely on nodal stage. While promising, the limited number of events and absence of external validation raise concerns about potential overfitting and generalizability. Mao et al. (2021) [83] conducted a larger, multicenter retrospective study involving 304 patients with ER-positive, LN-negative invasive breast cancer to predict recurrence risk based on Oncotype DX (the 21- gene recurrence risk score test). Their radiomics model, based on preoperative MM, achieved AUCs of 0.88 in the internal cohort and 0.84 in an external validation set. While lower than Jiang et al.’s results, the inclusion of an independent external test set makes these findings very significant. Additionally, the study incorporated multiple feature selection and classifier strategies, improving methodological transparency. Nonetheless, the retrospective design, and limited discussion on feature stability across scanners and acquisition protocols suggest that further standardization is needed before real-world translation.

Ma et al. (2024) [84] explored prognosis prediction in a rare setting—phyllodes tumors, which account for less than 1% of breast tumors—using a multimodal radiomics model combining MM and MRI. Their pipeline achieved an AUC of 0.95 and accuracy of 0.923 for disease-free survival prediction after surgery. While phyllodes tumors are typically understudied in AI literature due to their low prevalence, this work demonstrates the feasibility of predictive modeling even in rare breast cancer subtypes. Despite the encouraging results, the model’s generalizability remains uncertain due to the small cohort (*n* = 131) and the uniqueness of the tumor type, which may limit direct extrapolation to more common breast cancers. Nonetheless, the study marks an important step toward expanding AI applicability beyond the dominant focus on invasive ductal carcinoma.

Yu et al. (2021) [85] investigated the potential of MM-based radiomics to predict tumor-infiltrating lymphocyte levels, achieving moderate performance (AUC = 0.79; accuracy = 0.639). Despite that, the study addresses an innovative and clinically relevant target, as tumor-infiltrating lymphocytes reflect tumor immunogenicity and are increasingly recognized as predictive biomarkers for immunotherapy response. By identifying imaging correlates of immune infiltration, the authors open avenues for integrating radiomics into immuno-oncology workflows. However, the relatively low accuracy highlights the complexity of capturing microenvironmental features from standard mammograms, which lack functional or contrast information. Future work could benefit from multimodal data fusion and inclusion of histopathological or transcriptomic validation to improve robustness and clinical value.

In sum, CESM dominates the treatment response subfield, likely due to its functional imaging capabilities. Meanwhile, multimodal and longitudinal approaches are emerging as promising directions, but reproducibility, interpretability, and standardization remain key obstacles before AI-based prognostic tools can become routine in clinical oncology.

**Table 5:** Studies using mammography-based and tomosynthesis-based imaging for treatment response assessment and prognosis prediction in breast cancer.

Study	Clinical Target	Modality	AI Approach	No. of Cases	Dataset	Performance Metric
Jiang et al., 2020 [82]	Prognostic/recurrence prediction	MM	TradRad	200 patients	private	C-index = 0.944 (95% CI, 0.883–1.004) for predicting iDFS (invasive disease-free survival)
Wang et al., 2021 [86]	pCR to neoadjuvant chemotherapy	CESM	TradRad	117 patients	private	AUC = 0.81 (95% CI, 0.575–0.948), Acc = 0.80
Mao et al., 2021 [83]	Prognostic/recurrence prediction	MM	TradRad	304 patients	private	AUC = 0.88 (95% CI, 0.75–1.00) internal test set AUC = 0.84 (95% CI, 0.69–0.99) external test set
Yu et al., 2021 [85]	Tumor-infiltrating lymphocyte	MM	TradRad	121 patients	private	AUC = 0.79 (95% CI, 0.615–0.964), Acc = 0.639
Skarping et al., 2022 [87]	pCR to neoadjuvant chemotherapy	MM	End-to-end DL	453 patients	private	AUC = 0.71 (95% CI, 0.53–0.90)
Mao et al., 2022 [79]	pCR to neoadjuvant chemotherapy	CESM	TradRad	118 patients	private	AUC = 0.85 (95% CI, 0.72–0.98)
Zhang et al., 2023 [88]	pCR to neoadjuvant chemotherapy	CESM	TradRad	118 patients	private	AUC = 0.790 (95% CI, 0.554–0.952), Acc = 0.861
Xing et al., 2024 [80]	pCR to neoadjuvant chemotherapy	CESM	End-to-end DL	265 patients	private	AUC = 0.95 (95% CI, 0.847–1.0), Acc = 0.94
Förnvik et al., 2024 [81]	pCR to neoadjuvant chemotherapy	BT (volume-based)	End-to-end DL	149 patients	private	AUC = 0.83 (95% CI, 0.63–1.00)

**Table 6:** Studies using multimodal imaging approaches (including mammography and tomosynthesis) for treatment response assessment and prognosis prediction in breast cancer.

Study	Clinical Target	Modality	AI Approach	No. of Cases	Dataset	Performance Metric
Han et al., 2024 [89]	Prognostic/recurrence prediction	MM + US	End-to-end DL	1242 patients	private	AUC = 0.739 (95% CI, 0.608–0.871), Acc = 0.798 (95% CI, 0.797–0.800) disease-free survival
Ma et al., 2024 [84]	Prognostic/recurrence prediction	MM + MRI	TradRad	131 patients	private	AUC = 0.95 (95% CI, 0.92–0.98), Acc = 0.923 disease-free survival
Cai et al., 2024 [90]	pCR to neoadjuvant chemotherapy	BT (slice-based) + US	TradRad	720 patients	private	AUC = 0.81 (95% CI, 0.75–0.87), Acc =0. 764

## 5. Conclusions

This review highlights how AI, through radiomics and DL, is reshaping breast cancer characterization using MM and BT. From hormone receptor status to recurrence prediction, AI models have demonstrated the ability to non-invasively extract biological and clinical information directly from standard imaging. Radiomics remains widely used, particularly in contrast-enhanced spectral mammography (CESM), due to its interpretability and lower data requirements. DL strategies, especially end-to-end CNNs, are gaining momentum in MM, driven by larger datasets and reduced reliance on manual annotations.

A clear trend emerges: multimodal and weakly supervised approaches, integration of peritumoral regions, and temporal modeling are showing promise across multiple clinical targets. However, challenges persist. Most studies are retrospective, monocentric, and based on private data, which hampers reproducibility and comparison. Of the 81 studies included, only 6 used public or semi-public datasets, limiting transparency and external benchmarking. Furthermore, several studies address multiple clinical targets simultaneously, reflecting the feasibility of comprehensive, multiparametric models.

Beyond the limited availability of multicentric and public datasets, additional methodological challenges continue to hinder clinical translation. The variability in biomarker assessment—such as differences in immunohistochemical thresholds for ER, PR, HER2, and Ki-67—introduces inconsistencies in the ground truth used to train AI models. Many studies also face class imbalance, with underrepresented subtypes such as HER2-positive and triple-negative cancers reducing model generalizability. Moreover, segmentation strategies, image preprocessing and feature extraction methods remain heterogeneous across studies, complicating reproducibility and cross-cohort comparison. Addressing these limitations will require stronger harmonization of imaging and annotation protocols, transparent sharing of data and code, and larger, prospectively collected cohorts to support the development of reliable and clinically applicable models.

No filter was applied to the publication year during article selection, but all relevant studies were published between 2018 and 2025, underscoring the recent and fast-evolving nature of this field. Despite the growing number of publications, robust clinical integration of AI tools remains limited by the lack of external validation, absence of standardized image acquisition and annotation protocols, and variable outcome definitions.

## Abbreviations

The following abbreviations are used in this manuscript:

MM	mammography
BT	breast tomosynthesis
US	breast ultrasound
MRI	magnetic resonance imaging
AI	artificial intelligence
CESM	contrast-enhanced spectral mammography
DL	deep learning
CNN	convolutional neural networks
ROI	region of interest
ALN	axillary lymph node
pCR	pathological complete response
ER	estrogen receptor
PR	progesterone receptor
HER2	human epidermal growth factor receptor 2
IHC	immunohistochemistry
TN	triple-negative
DCIS	ductal carcinoma in situ
